# Retraction Note: Impact of the third wave of the COVID-19 pandemic and interventions to contain the virus on society and patients with kidney disease in Cambodia

**DOI:** 10.1186/s41100-022-00416-5

**Published:** 2022-06-15

**Authors:** Tam Nov, Toru Hyodo, Yukie Kitajima, Kenichi Kokubo, Toshihide Naganuma, Haruki Wakai, Akihiro Yamashita, Elin Phon, Hideki Kawanishi

**Affiliations:** 1 International University (IU), Phnom Penh, Cambodia; 2 Cambodia Association of Nephrology (CAN), Phnom Penh, Cambodia; 3 NGO Ubiquitous Blood Purification International (NGO UBPI), Yokohama, Japan; 4 Japanese Assistance Council for Establishing Dialysis Specialists’ System in Cambodia, Yokohama, Japan; 5 Department of Urology, Osaka City University, Osaka, Japan

## Retraction of: Ren Replace Ther (2021) 7:53 https://doi.org/10.1186/s41100-021-00372-6

The Editor-in-Chief has retracted this article. After publication, concerns were raised regarding the ethics approval for this study. The authors have confirmed that approval from the National Ethics Committee for Health Research in Cambodia was not sought prior to the study.

All authors agree to this retraction.

